# Age as a decisive factor in general anaesthesia use in paediatric proton beam therapy

**DOI:** 10.1038/s41598-020-72223-z

**Published:** 2020-09-15

**Authors:** Yuzo Shimazu, Rie Otsuki, Masao Murakami, Akio Konishi, Keiichi Kan, Ichiro Seto, Hisashi Yamaguchi, Masaharu Tsubokura, Hisashi Hattori

**Affiliations:** 1 Department of Anesthesiology, Southern Tohoku Research Institute for Neuroscience Southern TOHOKU General Hospital, Koriyama City, Fukushima 963–8563 Japan; 2 Southern TOHOKU Proton Therapy Center, Southern Tohoku Research Institute for Neuroscience Southern TOHOKU General Hospital, Koriyama City, Fukushima 963–8562 Japan; 3 Department of Public Health, Fukushima Medical University School of Medicine, Fukushima City, Fukushima 960–1295 Japan

**Keywords:** Paediatric research, Medical research

## Abstract

Proton therapy for paediatric cancer patients is an effective treatment; however, young children have may have difficulties staying still during irradiation. This study investigated the indication of general anaesthesia in paediatric proton therapy. Background information and anaesthesia/treatment protocols were retrospectively extracted from the medical records of cancer patients under 15 years who underwent proton therapy at Southern TOHOKU General Hospital, Fukushima, Japan between April 2016 and December 2018. The anaesthesia and non-anaesthesia groups were compared to evaluate factors determining the need for general anaesthesia. Thirty-two patients who received 285 irradiations were analysed. The median age was 5 years old (range: 1–15), and 13 patients (40.6%) were female. Twelve (37.5%) patients received general anaesthesia. In the general anaesthesia group, airway management using a laryngeal mask was performed in 11 patients (91.6%). Patient age was significantly lower in the general anaesthesia group than in the non-anaesthetised group (p < 0.001). Considering all background factors, only age was strongly associated with anaesthesia in the univariate logistic regression model (odds ratio 0.55 [95% confidence interval 0.35–0.86]; *P* < 0.01). Thus, age is one of the most important factors determining the need for general anaesthesia during proton therapy in children.

## Introduction

General anaesthesia is an essential measure when performing an invasive treatment, which involves putting the patient in a non-physiological state via administration of an anaesthetic. Subjects who undergo general anaesthesia range from prenatal neonates to elderly people over 100 years of age. More recently, the number of cases requiring general anaesthesia have risen with an increase in patients with cancer and cerebrovascular disease and with changes in disease structure. According to a 2008 report, more than 200 million operations under general anaesthesia were performed worldwide^1^. Under such circumstances the need for anaesthesia outside the operating room is rapidly increasing. Treatments requiring general anaesthesia, besides surgical operation, include cardiovascular catheterisation such as ablation, stent graft interpolation, endovascular treatment, radiological embolisation, gamma knife, and proton beam therapy (PBT)^2^. Performing general anaesthesia outside the operating room raises safety and quality issues due to the limited availability of equipment and anaesthesiologists, who might not be available in case of an emergency^3,4^.

Among advances in radiation therapy technology PBT has emerged as a promising treatment for cancer. The therapeutic effects of PBT in childhood cancer, especially for brain tumours and neuroblastoma, are high, and its application is on the rise worldwide^5–8^. Because PBT for children requires a longer immobilisation period during irradiation compared with general radiation therapy, general anaesthesia and sedation may be required for immobilisation^9^. Therefore, cooperation between radiologists and anaesthesiologists is important for patients who need general anaesthesia or sedation. However, since it is necessary to perform this procedure several times every day, it is important to properly select subjects considering the risks. While several researchers have reported on the safety and efficacy of general anaesthesia during PBT, there is a lack of evidence on the safety of repeated anaesthesia and sedation during PBT in children^10–12^. The reports lacked information on equipment standards used for safe general anaesthesia management, and there was limited information on how treatment choices were made at each facility.

In Japan, health insurance for PBT has been rapidly implemented for paediatric tumours covered by insurance since 2016 and head & neck cancers (excluding squamous cell tumours of the oral cavity or pharynx), prostate cancer, and bone and soft tissue sarcoma since 2018. Given that PBT has a high therapeutic effect (high local control rate and low damage to normal tissue) and is covered by insurance for childhood cancers, the use of PBT as an option for radiation therapy is expected to increase^13–15^.

The Southern TOHOKU Proton Therapy Center (Koriyama City, Fukushima Prefecture, Japan) was established in October 2008 as the first private particle therapy facility in Japan. The centre has performed PBT on a total of 5,000 people from October 2008 till August 2019. The requirement of sedation is discussed prior to the start of therapy for each patient to ensure proper treatment^16^. This situation enabled us to collect retrospective information regarding the treatment of these patients at Southern TOHOKU General Hospital to better understand which patients require sedation.

In order to examine the indications for general anaesthesia in paediatric PBT, information on patients under 15 years of age was extracted retrospectively (e.g. age, body weight, primary disease, radiation therapy, and general anaesthesia) and was compared between patients that required general anaesthesia and patients that did not require general anaesthesia.

## Methods

### Study setting

This study was a retrospective observational study conducted at the Southern TOHOKU Proton Therapy Center. The system (Mitsubishi Electric Corporation) consists of a synchrotron accelerator, two gantries and one fixed horizontal beamline. The synchrotron system can deliver passively scattered protons at a range of energies up to 235 meV.

### Patients and variables

We included paediatric patients (aged 15 years or younger) who underwent PBT at our hospital from April 2016 to December 2018. The patients were referred by the Department of Pediatric Oncology, Fukushima Medical University. Using the database of electronic medical records at our hospital, age, gender, body mass index (BMI), primary disease diagnosis, performance status (PS) (0–4), and details of treatment before PBT (chemotherapy, other radiotherapy, presence or absence of surgery) were extracted for each patient. Regarding treatment, information was extracted on the irradiation site, the irradiation dose, the number of irradiations, the number of irradiation days, and the presence or absence of anaesthesia (general anaesthesia including sedation or no anaesthesia).

### Anaesthesia management

While each patient received multiple treatments, the same anaesthetic (i.e. propofol and fentanyl) was used for each of their treatments. To determine whether general anaesthesia or sedation was necessary during PBT, an anaesthesiologist participated in CT imaging during treatment planning.

The anaesthesiologist would observe pretreatment CT scans to evaluate whether airway obstruction would occur when patients received propofol. The anaesthesiologist would then adjust the dose of propofol for the CT scans based on their experience. If the patient had airway obstruction during the CT scan, propofol boluses and fentanyl were administered, and a laryngeal mask airway (LMA) was placed at the time of the actual proton beam therapy.

### Analysis

In order to assess the indications for general anaesthesia in paediatric PBT, we performed the following two analyses:

1. Firstly, to identify differences in demographic and clinical characteristics between the anaesthesia and non-anaesthesia groups, these variables were compared between the two groups. Chi-square tests were used for the comparison of categorical variables and two-sample t-tests were used for the comparison of continuous variables.
2. Secondly, to identify the factors associated with an indication for general anaesthesia, we constructed a multivariate logistic regression model. The outcome variable was dichotomous (i.e., the presence of general anaesthesia (including sedation) versus no anaesthesia). Variables considered in the model were: age, sex, disease (brain tumours or other), PS (0–1 or >  = 2), pre-treatment, and the number of irradiation sites (single or multiple sites). The variable selection was based on univariate analyses.

All analyses were performed using Stata/IC version 15.0 (StataCorp LP). P-values < 0.05 were considered statistically significant.

### Ethics

Inclusive consent was obtained from the parents/guardians of patients. This study was approved by the Institutional Review Board of the Southern Tohoku Research Institute for Neuroscience, Southern TOHOKU General Hospital (Authorisation number 378). All methods in the present study were performed in accordance with the STROBE Statement.

## Results

Thirty-two patients from our hospital were included in the final analysis. Patients’ characteristics are shown in Table 1. Age and BMI were based on records at the start of treatment. The median age was 5 years (range: 1–15 years) and 19 patients were male (59.4%) and 13 (40.6%) were female. The median BMI was 16.7 (range: 13.3–32.0) kg/m^2^. Thirteen patients had brain tumours, which included 5 with medulloblastoma, 4 with glioma, 2 with ependymoma, and 1 patient each with germinoma and intraventricular tumour. Nineteen patients had tumours other than brain tumours, which included 9 with neuroblastoma, 3 with rhabdomyosarcoma, 2 with AML extramedullary and 1 patient each with B cell lymphoma, Ewing’s sarcoma, myofibroblastoma, buccal mucosal schwannoma, and retroperitoneal tumour. There was no difference in the mean age between patients with brain tumours and those without brain tumours (p = 0.67).

**Table 1:** Patient characteristics.

		Overall	General anaesthesia		p value *
			Yes	No	
		n = 32	12	20	
Age (years)	Median, range	5 (1–15)	3 (1–7)	8.5 (3–15)	P < 0.001
Age category	0–3	10	6 (60%)	4 (40%)	P < 0.01
	4–7	11	6 (54.5%)	5 (45.5%)	
	8–15	11	0 (0%)	11 (100%)	
Sex	Male	19	8 (42.1%)	11 (57.9%)	0.52
	Female	13	4 (30.8%)	9 (69.2%)	
BMI (kg/m^2^)	Median, range	16.7 (13.3–32.0)	16.3 (13.6–20.3)	17.5 (13.3–32.0)	0.20
Brain tumour	Medulloblastoma	5	3 (60%)	2 (40%)	0.78
	Glioma	4	1 (25%)	3 (75%)	
	Other brain tumours	4	1 (25%)	3 (75%)	
	Ependymoma	2	0 (0%)	2 (100%)	
	Germinoma	1	1 (100%)	0 (0%)	
	Intraventricular tumour (Teratoma)	1	0 (0%)	1 (100%)	
Non-brain tumour	Neuroblastoma	9	5 (55.6%)	4 (44.4%)	
	Rhabdomyosarcoma	3	1 (33.3%)	2 (66.7%)	
	AML extramedullary	2	1 (50%)	1 (50%)	
	Other	5	0 (0%)	5 (100%)	
Pre-treatment	Radiation	20	7 (35%)	13 (65%)	0.71
	Chemotherapy	11	4 (36.4%)	7 (63.6%)	0.92
	Operation	19	9 (47.4%)	10 (52.6%)	0.16
Irradiation site	Intracranial	16	7 (43.7%)	9 (56.3%)	0.47
	Head and neck	5	2 (40%)	3 (60%)	0.90
	Spinal cord	12	6 (50%)	6 (50%)	0.26
	Chest	3	0 (0%)	3 (100%)	0.16
	Abdomen and Retroperitoneum	11	5 (45.5%)	6 (54.6%)	0.50
	Other	2	1 (50%)	1 (50%)	0.71
Primary site of irradiation	Intracranial	13	6 (46.2%)	7 (53.8%)	0.58
	Head and neck	5	1 (20%)	4 (80%)	
	Chest	0	0 (0%)	0 (0%)	
	Spinal cord	1	0 (0%)	1 (100%)	
	Abdomen and Retroperitoneum	8	4 (50%)	4 (50%)	
	Other	5	1 (20%)	4 (80%)	
Dose (Gy)	Median, range	50.4 (19.8–66.0)	45.2 (19.8–61.2)	50.4 (19.8–66.0)	0.73
Treatment length (days)	Median, range	22.5 (10–33)	20.0 (10–33)	25.0 (11–33)	0.58
Adverse side effect		1	1 (8.3%)	0	

*P-values for chi-square test and two-sample t-tests for comparisons between general anaesthesia group and non-anaesthesia group.

As most patients received multiple anaesthetics, the median dose per treatment for each patient was calculated, and the median of the median dose of all patients was also calculated. The median PS before PBT was 1 (range: 0–4). The total number of irradiations was 285 and the median treatment duration was 22.5 days. The median irradiation dose was 50.4 (range: 19.8–66.0) Gy. While 13 patients were irradiated at multiple locations, the number of irradiation sites was 16 inside the cranium, 5 in the head and neck, 12 in the spinal cord, 3 in the chest, 11 in the abdomen and retroperitoneum, and 2 in other sites. The primary site of irradiation was 13 inside the cranium, 1 in the spinal cord, 5 in the head and neck, 8 in the abdomen and retroperitoneum, and 5 in other sites (including the femur or sacral bones). In the group who underwent general anaesthesia, patients were significantly younger than in the group without general anaesthesia (P < 0.001) (Table 1). There were no other statistical differences in demographics or clinical variables between the two groups. The oldest patient in the group who received anaesthesia was 7 years old. This 7-year-old patient with glioblastoma and autism was unable to stay still and underwent general anaesthesia. In contrast, the youngest patient in the group that did not receive anaesthesia was 3 years old. This 3-year-old patient was quiet and had a short treatment time and therefore, could be irradiated without general anaesthesia.

Regarding the method of anaesthesia, 12 patients (37.5%) underwent general anesthesia/sedation (Table 2). Airway management using LMA was performed in 11 patients (91.6%). Propofol alone was used in one patient. The median dose of propofol was 30 mg (range: 8–50 mg), the median dose of fentanyl was 30 mg (range: 0–72 mg), and the median concentration of sevoflurane was 1.5% (range: 0–3.0%). The median time to awakening from anaesthesia was 21 min (range: 5–52 min), and the median time in the treatment room was 45 min (range: 15–70 min) (Table 2).

**Table 2:** Characteristics of general anaesthesia/sedation.

Airway securing*		
Yes (using Laryngeal Mask)	11	91.7%
No	1	8.3%
Anaesthetic	Median	Range
Propofol dose (mg/kg)	2.31	1.57–3.88
Fentanyl dose (μg/kg)	2.34	0–3.85
Sevoflurane concentration (%)	1.5	0–3.0
**Adverse side effects (n = 1)**		
Body movement	0	0%
Hypoxia	0	0%
Nausea and vomiting	1	3.1%
Other	0	0
	Median	Range
Time to awakening (min)**	21	5–52
Irradiation time (min)	29	14–75
Time in the irradiation room (min)	40	25–79

*One patient who received general anaesthesia without a laryngeal mask airway was given the same doses of propofol for all of the multiple treatments.

**The time from the end of irradiation (end of anaesthesia) to when arousal or body movement is observed.

A logistic regression analysis identifying variables that predicted the use of general anaesthesia for PBT was performed. While each patient received multiple treatments, if general anaesthesia was indicated for a patient, they then received anaesthesia across all treatments. Results of the logistic regression analyses for the indication of general anaesthesia are shown in Table 3. Owing to a lack of significant variables and the limited number of cases, we also performed univariate logistic regression for each variable. Thus, Table 3 also presents the results of the univariate logistic regression models. We found a statistically significant association between age and an indication for general anaesthesia: odds ratio (OR) 0.55 [95% confidence interval (CI) 0.35–0.86] (*P* < 0.01). Other variables were not significantly associated with an indication for general anaesthesia.

**Table 3:** Results of univariate analyses: odds ratio for factors associated with general anaesthesia (95% confidence interval).

	Odds ratio	95% confidence interval
Age (years)	0.55	0.36−0.85*
Sex		
Male	1.00	
Female	0.61	0.14–2.71
Disease		
Brain tumours	1.00	
Other tumours	0.93	0.22–4.00
Primary site of irradiation		
Intracranial	1.00	
Other sites	1.86	0.43−7.98
Performance status		
0 − 1	1.00	
≧ 2	0.58	0.14–2.48
Irradiation site Single	1.00	
Multiple	3.27	0.73–14.6
Pre-treatment		
Chemotherapy −	1.00	
+		0.20−4.21
Operation −	1.00	
+	2.41	0.62−14.5
Radiation −	1.00	
+	0.75	0.17−3.28

*P value < 0.01.

Regarding side effects related to general anaesthesia, aspiration and vomiting after irradiation were observed in one (8.3%) out of 12 patients because of bulbar palsy caused by the underlying disease. Movement and severe hypoxemia during irradiation were not observed in any patients.

## Discussion

In recent years, the use of general anaesthesia has increased in and outside of the operating room because of an increase in patients with cancer and cerebrovascular disease and with changes in disease structure. While there are many reports on PBT showing good therapeutic results in cancer treatment^17,18^, an optimised treatment plan is required for highly accurate treatment. This requires time for proper alignment, immobilisation during irradiation, and general anaesthesia or sedation to suppress body movement. Given that general anaesthesia and sedation need to be performed several times daily, it is important to properly select subjects who are most in need of anaesthesia. While it has been considered safe for PBT to be performed under general anaesthesia in children, little information is available on the indications for general anaesthesia among patients who receive PBT.

This study has shown that age is one of the most important factors determining the need for general anaesthesia during PBT in children. According to our results, the median age was 3 years in the group in which general anaesthesia was performed, while the median age was significantly higher at 8.5 years in the group in which general anaesthesia was not performed. In particular, the oldest patient in the group who received anaesthesia was 7 years old, while the youngest patient in the group that did not receive anaesthesia was 3 years old. Vigneron et al.^10^ reported that children under 4 years of age required general anaesthesia during radiation therapy. On the other hand, McMullen et al.^11^ reported the need for general anaesthesia in almost all children under 3 years old, in about half of 7–8 years old, and in about 10% of 12 years or older. In addition, Owusu-Agyemang et al.^19^ reported the need for general anesthesia and sedation in 72% of patients under 4 years old, 45% of 4–6 years old, 15% of 7–10 years old, and 6% of 10 years or older. Our study has demonstrated statistically that age is the most important factor in paediatric proton therapy under general anesthesia, while Vigneron et al. and Owusu-Agyemang et al. only presented descriptive data. In the present study, there is a clear difference in the average age between the two groups, and there is a tendency similar to previous reports. Currently, there is a consensus on general anesthesia for children under 3 years of age during radiation therapy^12^. However, for children aged 7–8 years, the criteria for general anesthesia between institutions are ambiguous and there is no consensus. While age is an important factor in determining general anesthesia indications, Mizumoto et al.^20^ reported that even for children aged 4 to 6 years the irradiation time could be significantly shortened without general anesthesia by actively adjusting the treatment environment and performing interventions before PBT. In the future, it will be necessary to collect data across institutions and to develop consensus guidelines for the indication of general anesthesia/sedation.

The present study also showed that the safety of PBT under general anesthesia was sufficiently maintained in children under 15 years of age. Among the 12 patients irradiated under general anesthesia there was only 1 (8.3%) adverse effect of anesthesia. This rate of adverse side effects is similar to previous reports^21–24^. On the other hand, at the time of general anesthesia, it is necessary to discuss whether airway securing is necessary for proper safety. In fact, 91.6% of cases in this study required the insertion of a LMA, and during irradiation anesthesia management that preserved spontaneous breathing by sevoflurane inhalation was the first choice. As for patients treated in our hospital, it was found that propofol alone could be used to reduce physical activity without the need for LMA management in one case. In a previous report, Owusu-Agyemang et al.^12^ performed a total of 9,430 proton treatments in 340 patients and performed intravenous anesthesia (sedation) only with propofol in all patients. As a result, although LMA was inserted in 2 cases due to a decrease in oxygen saturation, it was reported that 97.3% of cases could be managed without securing the airway. Therefore, it is possible that anesthesia and sedation can be performed without inserting a LMA and securing the airway. Future research is necessary to assess the difference in patient safety depending on the presence or absence of a secured airway.

Although it may be possible to perform PBT with general anesthesia/sedation without securing the airway, there are problems with respiratory depression, apnoea, and airway obstruction due to tongue base depression^25^. It cannot be asserted whether airway patency can be maintained when propofol is administered in a sufficient amount to suppress movement. There are various ways to secure propofol management. Firstly, the use of an electroencephalogram monitor (BIS monitor)^26^ as an indicator of objective sedation during propofol administration may be effective, but it is desirable to develop new techniques and devices for evaluating airway patency in children with various responses to sedation. Secondly, regarding treatment and anaesthesia equipment, during PBT the treatment room and control room are completely isolated as in the case of a MRI examination. Therefore, it is essential for management to install equipment for patient monitoring and vital equipment to measure tidal volume and respiratory rate accurately, including an exhaled carbon dioxide monitor^27–29^. Adequate staffing and up-to-date education of doctors and other medical staff are also desired. Thirdly, there are no clear safety standards for personnel, monitors, and facilities during radiation therapy and conditions vary among facilities. Although the costs of investment in safety equipment vary, it is desirable to develop common guidelines for safety equipment.

This study should be interpreted with several limitations in mind. This study is a retrospective study at a single institution, with a small number of registered cases, and anaesthesia data and nursing records were extracted from an electronic medical record system in which some data was missing.

This retrospective observational study has shown that age is one of the most important factors determining the need for general anesthesia for PBT in children. While the safety of PBT under general anesthesia was sufficiently maintained in children under 15 years, in order to perform PBT with general anesthesia/sedation without securing the airway it will be necessary to collect data across institutions and to develop a consensus guideline for the indication of general anesthesia/sedation.

## Abbreviations

PBT: Proton beam therapy
LMA: Laryngeal mask airway

## References

[1] Weiser TG (2008). An estimation of the global volume of surgery: A modelling strategy based on available data. Lancet (London, England).

[2] Fuchs-Buder T, Settembre N, Schmartz D (2018). Hybrid operating theater. Anaesthesist.

[3] Choi JW, Kim DK, Lee SH, Shin HS, Seong BG (2018). Comparison of safety profiles between non-operating room anesthesia and operating room anesthesia: A study of 199,764 cases at a Korean tertiary hospital. J. Korean Med. Sci..

[4] Miller D, Miller R (2005). Anesthesia, sixth edition.

[5] Baliga S, Yock TI (2019). Proton beam therapy in pediatric oncology. Curr. Opin. Pediatr..

[6] Vogel J (2018). Proton therapy for pediatric head and neck malignancies. Pediatr. Blood Cancer.

[7] Glaser A, Nicholson J, Taylor R, Walker D (2014). Childhood cancer and proton beam therapy. BMJ.

[8] Frei-Welte M, Weiss M, Neuhaus D, Ares C, Mauch J (2012). Pediatric anesthesia for proton radiotherapy: Medicine remote from the medical centre. Anaesthesist.

[9] McFadyen JG, Pelly N, Orr RJ (2011). Sedation and anesthesia for the pediatric patient undergoing radiation therapy. Curr. Opin. Anaesthesiol..

[10] Vigneron C (2013). General anesthesia in pediatric radiotherapy. Cancer Radiother..

[11] McMullen KP, Hanson T, Bratton J, Johnstone PA (2015). Parameters of anesthesia/sedation in children receiving radiotherapy. Radiat. Oncol..

[12] Owusu-Agyemang P (2014). Non-invasive anesthesia for children undergoing proton radiation therapy. Radiother. Oncol..

[13] Sakurai H, Ishikawa H, Okumura T (2016). Proton beam therapy in Japan: Current and future status. Jpn. J. Clin. Oncol..

[14] Association for Nuclear Technology in Medicine. Facilities of ion beam radiation therapy in Japan. https://www.antm.or.jp/05_treatment/04.html (23 April 2020, date last accessed)

[15] Japanese Society for Radiation Oncology. *List of diseases where radiation therapy is recommended and covered by health insurance (March 2018) (in Japanese)* https://www.jastro.or.jp/medicalpersonnel/particle_beam/2018/03/post-10.html. (April 2020, date last accessed).

[16] Shimazu Y, Hattori H, Adachi K, Otsuki R, Konishi A (2018). Approach of pediatric proton therapy with sevoflurane general anesthesia. J. Clin. Anesth..

[17] Mizumoto M (2017). Long-term follow-up after proton beam therapy for pediatric tumors: A Japanese national survey. Cancer Sci..

[18] Journy N (2019). Patterns of proton therapy use in pediatric cancer management in 2016: An international survey. Radiother. Oncol..

[19] Owusu-Agyemang P (2016). A multi-institutional pilot survey of anesthesia practices during proton radiation therapy. Pract. Radiat. Oncol..

[20] Mizumoto M (2015). Preparation of pediatric patients for treatment with proton beam therapy. Radiother. Oncol..

[21] Verma V (2016). Anesthesia complications of pediatric radiation therapy. Pract. Radiat. Oncol..

[22] Gonzalez LP (2012). Anesthesia-related mortality in pediatric patients: A systematic review. Clinics (Sao Paulo, Brazil).

[23] Grebenik CR, Ferguson C, White A (1990). The laryngeal mask airway in pediatric radiotherapy. Anesthesiology.

[24] Buchsbaum JC (2013). Repetitive pediatric anesthesia in a non-hospital setting. Int. J. Radiat. Oncol. Biol. Phys..

[25] Khurmi N, Patel P, Koushik S, Daniels T, Kraus M (2018). Anesthesia practice in pediatric radiation oncology: Mayo clinic Arizona's experience 2014–2016. Paediatr. Drugs.

[26] Kamata K (2011). Initial experience with the use of remote control monitoring and general anesthesia during radiosurgery for pediatric patients. Pediatr. Neurosurg..

[27] Japanese Society of Pediatrics, Japanese Society of Pediatric Anaesthesia and Japanese Society of Pediatric Radiology.* Joint recommendations for sedation during MRI examination. (in Japanese)* https://www.jpeds.or.jp/modules/guidelines/index.php?content_id=33 (23 April 2020, date last accessed).

[28] Godwin SA (2014). Clinical policy: Procedural sedation and analgesia in the emergency department. Ann. Emerg. Med..

[29] Whitaker DK, Benson JP (2016). Capnography standards for outside the operating room. Curr. Opin. Anaesthesiol..

